# Premorbid beta blockade in sepsis is associated with a lower risk of a lactate concentration above the lactate threshold, a retrospective cohort study

**DOI:** 10.1038/s41598-022-25253-8

**Published:** 2022-12-02

**Authors:** Liam Schneider, Debra Chalmers, Sean O’Beirn, Miles Greenberg, Grant Cave

**Affiliations:** 1 Hawkes Bay Hospital Intensive Care Unit, Hastings, New Zealand; 2 University of Notre Dame, Freemantle, Australia

**Keywords:** Biomarkers, Medical research

## Abstract

Sepsis and septic shock represent a significant worldwide mortality burden. A lactate greater than 4 mmol/L is associated with increased mortality in septic patients. This is the concentration at the “lactate threshold” where serum lactate concentrations rise markedly with increased workload in exercise. Hyperlactatemia in both sepsis and exercise is contributed to by adrenergic agonism which stimulates aerobic glycolysis, increasing lactate production and decreasing lactate clearance. Our hypothesis is that in patients with sepsis, treatment with beta blockers in the community will be associated with a lower probability of initial lactate ≥ 4 mmol/L. This was single centre retrospective cohort study. We used an in-house SQL Database for all admissions to ICU/HDU for the 2017–2020 calendar years. The dataset was filtered for an APACHE III Diagnosis of sepsis. T-tests were used for continuous data, Chi squared and Fisher’s exact test were used as appropriate to compare proportions. Logistic regression was used to investigate covariate effects. Of the 160 patient records analysed, 49 were prescribed beta blockers. A greater proportion of patients not prescribed beta blockers in the community had a first lactate ≥ 4 mmol/L (p = 0.049). This was robust to regression analysis. There was no difference in the proportion of patients with lactate ≥ 2 mmol/L (p = 0.52). In our cohort patients previously prescribed beta blockers were less likely to have a lactate of ≥ 4 mmol/mL. This supports the proposed mechanism that treatment with beta blockers increases the lactate threshold in sepsis. Further study is warranted.

## Introduction

Sepsis is a major global health issue accounting for 20% of global mortality and 11 million deaths in 2017^1^. Sepsis represents a significant proportion of intensive care unit (ICU) caseload^2^. Hyperlactatemia in sepsis and septic shock correlates with the severity of sepsis and associated mortality^3^. Reduction in serial lactate concentrations is associated with improved outcomes^4,5^, while goal directed therapy targeting lactate clearance is part of the 2021 surviving sepsis guidelines^6^. Given the position serum lactate holds in the assessment and management of sepsis, any premorbid factors which impact on lactate concentrations are of potential interest to the treating clinician.

### Lactate kinetics in health and the lactate threshold

**Figure 1:**
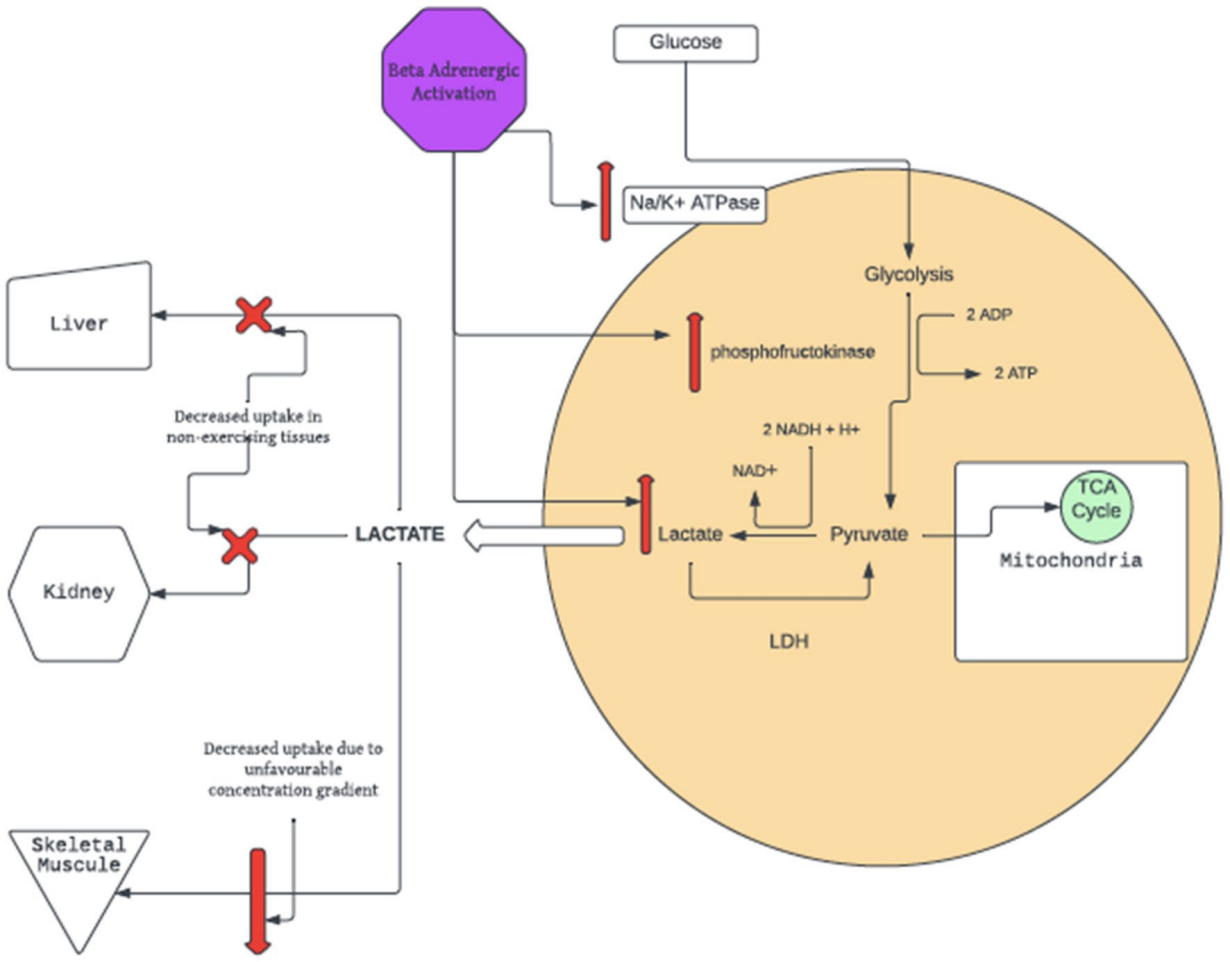
The proposed effect of beta adrenergic stimulation on lactate metabolism.

Lactate is produced from pyruvate during glycolysis. This reaction regenerates NAD+ for the production of adenosine triphosphate (ATP)^7^. Lactate is metabolised via gluconeogenesis in liver and kidney and oxidation in skeletal muscles^8–10^. At rest, lactate clearance is evenly distributed between these two mechanisms; during moderate to high exercise 60–80% of lactate clearance occurs in skeletal muscle^11^. The lactate threshold refers to the level of exercise intensity at which serum lactate accumulation rapidly increases^12^. This has been attributed to anaerobic metabolism and/or to an imbalance between lactate production and lactate clearance in the absence of tissue hypoxia^13,14^. The lactate threshold can be taken as 4 mmol/L with reasonable accuracy^15^. This threshold in health has a correlate in critical illness, with a marked increased mortality in sepsis when initial lactate is greater than 4 mmol/L^16^. In healthy subjects exercising at the lactate threshold, the amount of lactate metabolised is reduced compared to moderate exercise^17^. This suggests that part of the marked increase in lactate with increased exercise intensity above the lactate threshold is due to decreased metabolic clearance of lactate, an effect which may be mediated by beta agonism. Adrenergic stimulation of phosphofructokinase and Na/K ATPase result in increased conversion of glucose to pyruvate and cytosolic ATP to ADP, respectively. This increases production of lactate from pyruvate and ADP via lactate dehydrogenase in the cellular cytosol, resulting in decreased uptake of lactate by non-exercising tissues under adrenergic stimulation. This proposed mechanism is illustrated below in Fig. 1.

This physiology in health could also occur in sepsis—an adrenergically mediated decrease in cellular metabolism of lactate could hypothetically account for part of the hyperlactataemia in sepsis. Any such effect (and the effect of its blockade) may be more marked near the lactate threshold of 4 mmol/L.

### Hyperlactatemia in sepsis

Hypoperfusion and the resultant tissue hypoxia is traditionally held to explain the hyperlactatemia seen in sepsis^7,18,19^. Studies measuring partial pressures of oxygen in septic patients have however not demonstrated tissue hypoxia^20–24^, which has led to the consideration of other mechanisms^7^.

Activation of beta-2 adrenergic receptors, stimulated as part of a stress response is one such explanation^7,25^. This pathway has been experimentally blocked at various points, with a resultant reduction in lactate^22,26,27^. Esmolol infusion in septic patients was found to reduce lactate, and in beta blocker overdose a lower than expected lactate concentration is seen for the degree of haemodynamic compromise^28,29^.

Other mechanisms have been proposed. Impaired oxygen utilisation rather than inadequate oxygen delivery (DO_2_–VO_2_ mismatch) has been suggested, though there is little research correlating DO_2_–VO_2_ mismatch to lactate levels^30–33^. Gattinoni et al. hypothesised a combination of tissue hypoxia and inadequate oxygen utilisation as an explanation, finding that high lactate correlated in septic patients with either the highest or lowest central venous oxygen saturation (ScVO_2_)^34^. Other proposed mechanisms are the Warburg effect in immune activation and microcirculatory dysfunction^35–37^. It has been suggested that many or all of these suggested mechanisms play a role in hyperlactatemia in sepsis^38^.

### Premorbid beta blockade and lactate levels in sepsis

Five observational studies have assessed the effect of pre-morbid beta-blockade on lactate levels in patients presenting with sepsis^39–43^. Three trials found a significant reduction in lactate levels with beta-blockade^39,41,43^, while two showed no difference^40,42^. A recent meta-analysis of these studeis found lactate levels to be lower in patients on beta-blockers^44^.

**Figure 2:**
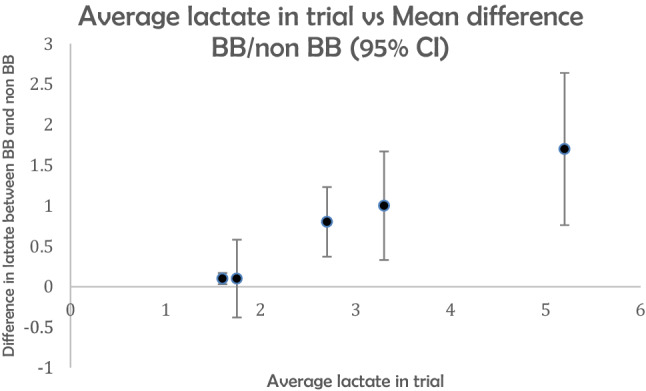
Average lactate in individual trials vs. difference in mean lactate for those prescribed and not previously prescribed beta blockers.

If the effect of beta blockade is mediated by an alteration of the lactate threshold, the effects on serum lactate would be expected to be greater in patient cohorts with higher lactates. Figure 2 represents data from these five studies.

All these trials have limitations—the majority of studies are retrospective observational in design; only one study was multicenter; two trials looked at lactate concentrations as a secondary outcome; inclusion criteria and sepsis definitions were variable between studies as was the timing of lactate measurement^39–43^. However, the pattern of data from the studies supports the theory that the effect of beta blockers on lactate is more pronounced in populations with higher lactate levels.

### Effect of premorbid beta-blockers on mortality

Pre morbid beta blockers may confer a mortality benefit in patients presenting with sepsis^40,43–47^, however the evidence is not homogenous^48,49^. The use of ultra-short acting beta blockers infusions in patient admitted with sepsis has shown a mortality benefit^28,50–56^. A meta-analysis including ten studies found premorbid beta blockade was associated with lower short term mortality in patients admitted with sepsis^44^*.* The proposed mechanisms of a mortality benefit include direct and indirect cardio protective effects; enhanced microvascular circulation due to reduction in coagulopathy and indirect immune modulatory effects^42,43,47,48^. In this regard evidence of a potentially beneficial effect on lactate metabolism of beta blockade would be of interest.

### Aim

To assess whether previous beta blocker prescription affected the probability that the first lactate in patients admitted from the Emergency Department to our intensive care unit with sepsis was above the lactate threshold.

### Hypothesis

The hypothesis was that in patients admitted with sepsis, treatment with beta blockers in the community will be associated with a lower probability of a lactate ≥ 4 mmol/L.

## Methods

### Setting

This was a retrospective cohort study conducted in the intensive care unit (ICU) of the Hawkes Bay Fallen Soldiers Memorial Hospital in Hastings, New Zealand. The hospital has 364 beds and approximately 1000 ICU and High Dependency Unit (HDU) admissions a year. The unit can provide mechanical ventilation and continuous renal replacement therapy, and cares for both adult and paediatric patients with medical and surgical conditions. Approval for the audit was granted by the Hawkes Bay DHB audit registration committee and the Northern B Health and Disability Ethics Committee of New Zealand. As only de-identified date was used a consent waiver was given by both committees. All research was performed in accordance with relevant guidelines/regulations. We used an In-house SQL Database that tracked all admissions to ICU/HDU for the 2017–2020 calendar years, which also allows collection of the ANZICS CORE Dataset. This was then filtered by a diagnosis of sepsis. A keyword search of free-text fields that supplemented the APACHE III Diagnosis that contained terms such as “Sepsis”, or “Septic Shock”, was carried out to identify those patients admitted with a co-diagnosis of sepsis. The data was then reviewed, and all duplicate or non-sepsis admissions were removed.

Initial serum lactate level at our centre was measured on an ABL 800 Flex blood gas analyser (Radiometer Medical ApS, Bronshoj, Denmark). Serum lactate was defined as the first lactate measured during an Emergency Department (ED) presentation. Only patients admitted directly from the ED were included. Serum lactate, current medications, presenting vital signs, illness severity scores, laboratory data and mortality outcome were extracted from patients’ electronic medical record and the unit’s clinical database.

### Inclusion

A single investigator (GC) blinded to beta blocker treatment status evaluated the electronic medical record to assess whether the clinical or microbiologic picture was consistent with infection.

### Exclusion

A single investigator (LS) reviewed the Emergency Department electronic medical record was reviewed and a qSOFA score calculated for each patient prior to evaluation of beta blocker status. Patients with qSOFA < 2 were excluded from analysis as patients with a qSOFA score < 2 are identified as low risk of sepsis^57^. Patients who did not have lactate measured were also excluded from analysis.

### Calculation of sample size

We used unpublished data from previous work where 25% fewer patients who were prescribed beta blockers in the community had an initial lactate > 4 mmol/L when compared with those not prescribed beta blockers (20% vs 45%). The study was powered under the assumptions that there would be the same proportion of beta blocked patients in the population and the proportions of patients with lactate > 4 mmol/L would be the same as in our previous work^41^. Under these assumptions, our study was > 80% powered at and alpha of 0.05 with 180 patient records included in the analysis. We anticipated that abstraction of four calendar years of data would provide these patient numbers.

### Statistical analysis

Our data were analysed using the Graphpad Prism version 9. Continuous data are presented as means with 95% confidence intervals. Proportions are presented as percentages with 95% confidence intervals. Students T-test was used for continuous data, Chi squared and Fisher’s exact test as appropriate to compare proportions. Logistic regression was used to investigate for significance and magnitude of covariate effects. Criterion for covariate entry into multiple regression modelling were a statistically significant difference in distribution of the variable between groups, an effect demonstrated in previously published work or a plausible covariate effect and a p value < 0.1 on univariate regression.

### Ethics approval and consent to participate

Approval for the use of de-identified data from this study was given by the Hawkes Bay hospital clinical audit committee and the Northern B Health and Disability Ethics Committee of New Zealand.

## Results

293 patient records were identified for audit. Of these, 129 were excluded for a qSOFA < 2 and a further 3 were excluded as the clinical situation and/or microbiology did not fit with the diagnosis of sepsis. One patient record of the remaining 161 did not have their lactate measured and was excluded from analysis.

### Baseline characteristics

The baseline characteristics for the 160 patients included in the analysis are presented in Table 1. At baseline the beta blocker group was older and had lower lactate than those not exposed to beta blockers. The site of sepsis by group is shown in Table 2. Fewer beta blocker patients had a respiratory source of sepsis. There was no statistically significant difference in mean lactate between those with a respiratory source of sepsis and those with other sites (mean difference 0.48, 95% CI − 1.04 to 2.0 mmol/L) nor between those with APACHE classified chronic cardiovascular disease (mean difference 0.08, 95% CI − 1.75 to 1.91 mmol/L).

**Table 1:** Baseline characteristics.

	Beta blocker	Non-beta blocker	p value for difference
Number (n)	49	111	
Male (%)	32 (65)	64 (58)	0.36
Age (years)	71	63	< 0.01
First lactate (mmol/L)	3.54	4.46	0.04
Lowest systolic blood pressure in ED (mmHg)	90	91	0.64
Lowest HR first 24 h in ICU	75.6	78	0.42
SaO_2_ (%)	92	90	0.74
Highest Creatinine first 24 h (mmol/L)	173	187	0.55
Lowest Haematocrit first 24 h	0.32	0.33	0.34
APACHE III score	75	70	0.31
Number qSOFA score 3 n(%)	7 (14)	16 (14)	0.98
Vasopressors used in ED, n (%)	24 (49)	63 (57)	0.36
Prescribed metformin, n (%)	14 (29)	22 (19)	0.22
Chronic cardiovascular disease (APACHE) n (%)	6 (12)	5 (5)	0.07
Chronic respiratory disease (APACHE) n (%)	1(2)	7(6)	0.67
Mortality, n (%)	8 (16)	17 (15)	0.87

**Table 2:** Site of sepsis by group.

Site of sepsis	Beta blocker, n (%)	Non-beta blocker, n (%)	p value
Respiratory	7(14)	32 (29)	0.05
Skin/soft tissue/joint	16 (33)	26 (23)	0.22
Genitourinary	12 (25)	18 (16)	0.22
Unknown	5 (10)	24 (22)	0.08
Biliary	5 (10)	3 (3)	n/s
Intra-abdominal	3 (6)	3 (3)	n/s
Cardiac	0 (0)	2 (2)	n/s
Vascular catheter	1 (2)	0 (0)	n/s
CNS	0 (0)	1 (1)	n/s

### Primary endpoint

**Figure 3:**
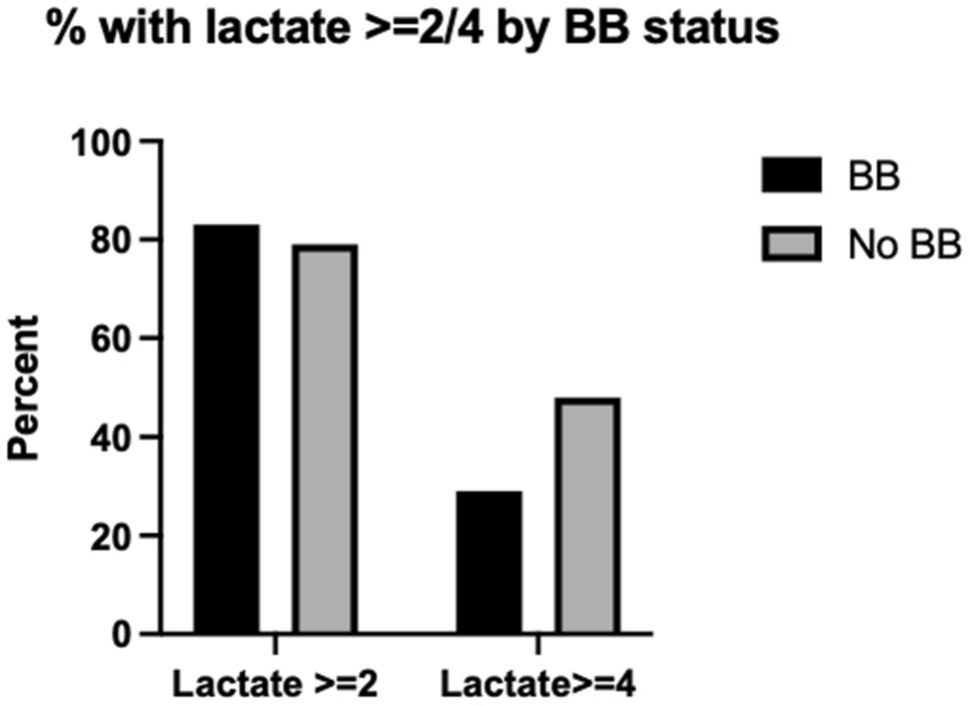
Percentages of those prescribed and not prescribed beta blockers with lactate greater than 2 and 4 mmol/L at presentation.

A greater proportion of patients not prescribed beta blockers in the community had a first lactate ≥ 4 mmol/L (48% of patients no beta blockers vs 29% prescribed beta blockers, p = 0.049). There was no significant difference in the proportion of patients with lactate ≥ 2 mmol/L (79% of non-beta blocker patients versus 83% of beta blocker patients, p = 0.52). These results are displayed graphically in Fig. 3.

### Covariates and regression analysis

Of the covariates in table 3 only APACHE 3 score was significantly correlated with lactate. Logistic regression was undertaken to evaluate the effect of covariates as per the analysis plan in the methods. The odds ratio for beta blocker prescription for first lactate being ≥ 4 mmol/L regressed for the covariates age, APACHE 3, metformin prescription, site of sepsis being respiratory and lowest haemotocrit (Hct Lo) in the first 24 h of ICU admission was 0.31 (0.13–0.71). Of note the upper bound of the 95% CI for the odds ratio was < 1. This finding was robust to regression with covariates individually, the removal from the model of age (which exhibited a linear correlation with Apache 3), and addition of lowest systolic blood pressure in the Emergency Department to the model.

**Table 3:** Covariate analysis.

Covariate	95% CI odds ratio, p value
Male sex	0.8–1.75 to 0.21, p 0.82
Age (years)	0.98–1.01, p 0.99
Lowest recorded SpO_2_ in ED (%)	0.96–1.07, p 0.36
Lowest haematocrit first 24 h	0.99 to 1.1, p 0.053
APACHE III score	1.013 to1.04, p < 0.001
Vasopressors used in ED	0.39 to 1.4, p 0.44
SBP in ED	0.96–1, p 0.11
Prescribed metformin	0.8–3.8, p 0.31
Site of sepsis respiratory	0.31–1.4, p = 0.6
Cardiovascular disease (APACHE)	0.35–4.4, p = 0.89

## Discussion

In this patient cohort pre-morbid beta blocker treatment was associated with a lower initial lactate, driven by a reduction in the proportion of patients with a lactate of ≥ 4 mmol/L. This effect was robust to regression analysis. There was no significant difference in the proportion of beta blocker/non beta blocker exposed patients with lactate ≥ 2 mmol/L. Extension from our findings holds obvious caveats in that our methodology permits identification of association only, and lactate threshold is a concept from exercise physiology that has not been proven to have an effect in the clinical context. Nonetheless, these findings provide inferential support for premorbid beta blockade reducing serum lactate in sepsis by increasing the proportion of patients below the concentration where lactate production and metabolism uncouple in response to metabolic stress. Restated, our findings offer support for the view that beta blockers increase the lactate threshold in sepsis.

This data fits with the pattern observed the previous five studies as demonstrated in Fig. 2, that the effect of premorbid beta-blockers on initial lactate was most significant in patient populations with higher mean lactate concentrations. The papers which found no effect analysed cohorts with an average initial lactate of ≤ 2 mmol/L^40,42^; we did not identify any association for premorbid beta blockade with the probability of lactate being ≥ 2 mmol/L. The three other studies which demonstrated an effect of beta blockade when looking at cohorts with higher average lactates^39,41,43^. Our proposed mechanism to explain the findings seen in our study, while hypothetical, offers a unifying explanation for the current heterogeneity of evidence in this area.

Our study has several limitations. The most significant is that seeking an association based on a hypothesized mechanism can establish the association while the mechanism remains hypothetical. While there is a markedly increased mortality with lactate ≥ 4 mmol/L in sepsis the lactate threshold is a concept proven in exercise physiology rather than established in clinical medicine. Our study design is a retrospective observational and as such can only demonstrate an association rather than prove causation for the effects of premorbid beta blockade on serum lactate concentrations. Additionally, our study is single centre creating limits on external validity. The mean initial lactate was 3.54 mmol/L for the beta blocked patients compared to 4.46 mmol/L in those not premorbidly prescribed beta blockade. Both concentrations are above a threshold which would trigger clinical action—viewed from this perspective the clinical significance of the concentration difference is uncertain. This study is not powered to assess whether statistically significant difference in lactates concentration affected clinical outcomes. A qSOFA score of ≥ 2 was used as part of the inclusion criteria. The qSOFA score has been found to have a decreased predictive value in ICU compared to SOFA score but a better predictive value outside ICU^57^. A flaw with the qSOFA is the potential for inter-user variability in the recording of scores particularly in the altered mental status variable. In addition, some of the cohort was excluded due to incomplete data from the Emergency Department admission. As with other studies, it is only possible to ascertain whether patients were prescribed beta blockers at the time of to their admission, the actual compliance in the cohort is unknown. The reason for beta blocker prescription is not available for this patient cohort and as such the role of any underlying cardiac dysfunction is difficult to quantify. There may also be an effect of other unmeasured variables such as amount of fluid resuscitation prior to initial lactate measurement. We abstracted patient data in blocks of calendar years and anticipated 4 years would provide 180 records for study. The 5% reduction in power from analysis of 160 subjects from this period did not result in a type I error—our results were positive. Underpowered studies which return positive results additionally tend to overestimate the magnitude of effect—a lower proportion of underpowered studies are expected to be positive with the tendency to exhibit more extreme results. This was again not the case in our work as the overall difference in proportion of patients with lactate ≥ 4 in was lower than that powered for (19% vs 25%).

Further research is recommended into the effect of beta blockade on the lactate threshold and its significance. Our group has commenced bench top mechanistic work in a prospective study of the effect esmolol infusions on lactate in animal models of sepsis aiming to further examine the proposed mechanism of the effect of beta blockade on lactate.

## Conclusion

In our cohort patients previously prescribed beta blockers presenting with sepsis were less likely to have a lactate of ≥ 4 mmol/ml. This is in keeping with the pattern of results seen in the current literature and supports the proposed mechanism that treatment with beta blockers increases the lactate threshold. Further study is warranted as such a mechanism could have clinical significance.

## Data Availability

The datasets used and/or analysed during the current study are available from the corresponding author on reasonable request.

## References

[1] World Health Organization. Global report on the epidemiology and burden of sepsis: Current evidence, identifying gaps and future directions. World Health Organization. (2020) [cited 2022 Jun 5]. https://apps.who.int/iris/handle/10665/334216.

[2] Fleischmann-Struzek C, Mellhammar L, Rose N, Cassini A, Rudd KE, Schlattmann P (2020). Incidence and mortality of hospital- and ICU-treated sepsis: Results from an updated and expanded systematic review and meta-analysis. Intensive Care Med..

[3] Shapiro NI, Howell MD, Talmor D, Nathanson LA, Lisbon A, Wolfe RE (2005). Serum lactate as a predictor of mortality in emergency department patients with infection. Ann. Emerg. Med..

[4] Nichol A, Bailey M, Egi M, Pettila V, French C, Stachowski E (2011). Dynamic lactate indices as predictors of outcome in critically ill patients. Crit. Care.

[5] Jansen TC, van Bommel J, Schoonderbeek FJ, Sleeswijk Visser SJ, van der Klooster JM, Lima AP (2010). Early lactate-guided therapy in intensive care unit patients. Am. J. Respir. Crit. Care Med..

[6] Evans L, Rhodes A, Alhazzani W, Antonelli M, Coopersmith CM, French C (2021). Surviving sepsis campaign: International guidelines for management of sepsis and septic shock 2021. Crit. Care Med..

[7] Garcia-Alvarez M, Marik P, Bellomo R (2014). Sepsis-associated hyperlactatemia. Crit. Care.

[8] Consoli A, Nurjhan N, Reilly JJ, Bier DM, Gerich JE (1990). Contribution of liver and skeletal muscle to alanine and lactate metabolism in humans. Am. J. Physiol..

[9] Bergman BC, Wolfel EE, Butterfield GE, Lopaschuk GD, Casazza GA, Horning MA (1999). Active muscle and whole body lactate kinetics after endurance training in men. J. Appl. Physiol. (1985).

[10] Bergman BC, Horning MA, Casazza GA, Wolfel EE, Butterfield GE, Brooks GA (2000). Endurance training increases gluconeogenesis during rest and exercise in men. Am. J. Physiol. Endocrinol. Metab..

[11] Mazzeo RS, Brooks GA, Schoeller DA, Budinger TF (1986). Disposal of blood [1–13C]lactate in humans during rest and exercise. J. Appl. Physiol. (1985).

[12] Ga, B. Anaerobic threshold: Review of the concept and directions for future research. *Med. Sci. Sports Exerc.* (1985) [cited 2022 Mar 21]. **17**(1). https://pubmed.ncbi.nlm.nih.gov/3884959/.

[13] Wasserman K, Koike A (1992). Is the anaerobic threshold truly anaerobic?. Chest.

[14] Brooks GA (1986). The lactate shuttle during exercise and recovery. Med. Sci. Sports Exerc..

[15] Heuberger JAAC, Gal P, Stuurman FE, de Keizer WASM, Miranda YM, Cohen AF (2018). Repeatability and predictive value of lactate threshold concepts in endurance sports. PLoS ONE.

[16] Bou Chebl R, El Khuri C, Shami A, Rajha E, Faris N, Bachir R (2017). Serum lactate is an independent predictor of hospital mortality in critically ill patients in the emergency department: A retrospective study. Scand. J. Trauma Resusc. Emerg. Med..

[17] Messonnier LA, Emhoff CAW, Fattor JA, Horning MA, Carlson TJ, Brooks GA (2013). Lactate kinetics at the lactate threshold in trained and untrained men. J. Appl. Physiol. (1985).

[18] Dellinger RP, Levy MM, Rhodes A, Annane D, Gerlach H, Opal SM (2013). Surviving sepsis campaign: International, 2012. Intensive Care Med..

[19] Sterling SA, Puskarich MA, Shapiro NI, Trzeciak S, Kline JA, Summers RL (2013). Characteristics and outcomes of patients with vasoplegic versus tissue dysoxic septic shock. Shock.

[20] Rosser DM, Stidwill RP, Jacobson D, Singer M (1995). Oxygen tension in the bladder epithelium rises in both high and low cardiac output endotoxemic sepsis. J. Appl. Physiol. (1985).

[21] VanderMeer TJ, Wang H, Fink MP (1995). Endotoxemia causes ileal mucosal acidosis in the absence of mucosal hypoxia in a normodynamic porcine model of septic shock. Crit. Care Med..

[22] Levy B, Gibot S, Franck P, Cravoisy A, Bollaert PE (2005). Relation between muscle Na+K+ ATPase activity and raised lactate concentrations in septic shock: A prospective study. Lancet.

[23] Boekstegers P, Weidenhöfer S, Kapsner T, Werdan K (1994). Skeletal muscle partial pressure of oxygen in patients with sepsis. Crit. Care Med..

[24] Sair M, Etherington PJ, Peter Winlove C, Evans TW (2001). Tissue oxygenation and perfusion in patients with systemic sepsis. Crit. Care Med..

[25] Revelly JP, Tappy L, Martinez A, Bollmann M, Cayeux MC, Berger MM (2005). Lactate and glucose metabolism in severe sepsis and cardiogenic shock. Crit. Care Med..

[26] McCarter FD, James JH, Luchette FA, Wang L, Friend LA, King JK (2001). Adrenergic blockade reduces skeletal muscle glycolysis and Na(+), K(+)-ATPase activity during hemorrhage. J. Surg. Res..

[27] Levy B, Desebbe O, Montemont C, Gibot S (2008). Increased aerobic glycolysis through beta2 stimulation is a common mechanism involved in lactate formation during shock states. Shock.

[28] Morelli A, Ertmer C, Westphal M, Rehberg S, Kampmeier T, Ligges S (2013). Effect of heart rate control with esmolol on hemodynamic and clinical outcomes in patients with septic shock: A randomized clinical trial. JAMA.

[29] Mégarbane B, Deye N, Malissin I, Baud FJ (2010). Usefulness of the serum lactate concentration for predicting mortality in acute beta-blocker poisoning. Clin. Toxicol. (Phila)..

[30] Ronco JJ, Fenwick JC, Tweeddale MG, Wiggs BR, Phang PT, Cooper DJ (1993). Identification of the critical oxygen delivery for anaerobic metabolism in critically ill septic and nonseptic humans. JAMA.

[31] Ronco JJ, Fenwick JC, Wiggs BR, Phang PT, Russell JA, Tweeddale MG (1993). Oxygen consumption is independent of increases in oxygen delivery by dobutamine in septic patients who have normal or increased plasma lactate. Am. Rev. Respir. Dis..

[32] Mira JP, Fabre JE, Baigorri F, Coste J, Annat G, Artigas A (1994). Lack of oxygen supply dependency in patients with severe sepsis. A study of oxygen delivery increased by military antishock trouser and dobutamine. Chest.

[33] Astiz ME, Rackow EC, Kaufman B, Falk JL, Weil MH (1988). Relationship of oxygen delivery and mixed venous oxygenation to lactic acidosis in patients with sepsis and acute myocardial infarction. Crit. Care Med..

[34] Gattinoni L, Vasques F, Camporota L, Meessen J, Romitti F, Pasticci I (2019). Understanding lactatemia in human sepsis. Potential impact for early management. Am. J. Respir. Crit. Care Med..

[35] Cheng SC, Joosten LAB, Netea MG (2014). The interplay between central metabolism and innate immune responses. Cytokine Growth Factor Rev..

[36] Charlton M, Sims M, Coats T, Thompson JP (2017). The microcirculation and its measurement in sepsis. J. Intensive Care Soc..

[37] Ince C (2005). The microcirculation is the motor of sepsis. Crit. Care..

[38] Gutierrez G, Wulf ME (1996). Lactic acidosis in sepsis: A commentary. Intensive Care Med..

[39] Contenti J, Occelli C, Corraze H, Lemoël F, Levraut J (2015). Long-term β-blocker therapy decreases blood lactate concentration in severely septic patients. Crit. Care Med..

[40] Chan JZW, Tan JH, Lather KS, Ng AJY, Ong Z, Zou X (2020). Beta-blockers’ effect on levels of lactate in patients with suspected sepsis—The BeLLa study. Am. J. Emerg. Med..

[41] Pham D, Ward H, Yong B, Mahendra Raj J, Awad M, Harvey M (2021). Is lactate lower in septic patients who are prescribed beta blockers? Retrospective cohort study of an intensive care population. Emerg. Med. Australas..

[42] Tan K, Harazim M, Simpson A, Tan YC, Gunawan G, Robledo KP (2021). Association between premorbid beta-blocker exposure and sepsis outcomes-the beta-blockers in European and Australian/American Septic Patients (BEAST) Study. Crit. Care Med..

[43] Kuo MJ, Chou RH, Lu YW, Guo JY, Tsai YL, Wu CH (2021). Premorbid β1-selective (but not non-selective) β-blocker exposure reduces intensive care unit mortality among septic patients. J. Intensive Care.

[44] Hasegawa D, Sato R, Prasitlumkum N, Nishida K (2021). Effect of premorbid beta-blockers on mortality in patients with sepsis: A systematic review and meta-analysis. J. Intensive Care Med..

[45] Guz D, Buchritz S, Guz A, Ikan A, Babich T, Daitch V (2021). β-blockers, tachycardia, and survival following sepsis: An observational cohort study. Clin. Infect. Dis..

[46] Macchia A, Romero M, Comignani PD, Mariani J, D’Ettorre A, Prini N (2012). Previous prescription of β-blockers is associated with reduced mortality among patients hospitalized in intensive care units for sepsis. Crit. Care Med..

[47] Singer KE, Collins CE, Flahive JM, Wyman AS, Ayturk MD, Santry HP (2017). Outpatient beta-blockers and survival from sepsis: Results from a national cohort of Medicare beneficiaries. Am. J. Surg..

[48] Arnautovic, J., Mazhar, A., Souther, B., Mikhijan, G., Boura, J., Huda, N. Cardiovascular factors associated with septic shock mortality risks. *Spartan Med. Res. J*. [cited 2022 Feb 22]; **3**(1). https://www.ncbi.nlm.nih.gov/pmc/articles/PMC7746094/. 10.51894/001c.6516 PMC7746094 33655132

[49] DeMott JM, Patel G, Lat I (2018). Effects of chronic antihypertensives on vasopressor dosing in septic shock. Ann. Pharmacother..

[50] Hasegawa D, Sato R, Prasitlumkum N, Nishida K, Takahashi K, Yatabe T (2021). Effect of ultrashort-acting β-blockers on mortality in patients with sepsis with persistent tachycardia despite initial resuscitation: A systematic review and meta-analysis of randomized controlled trials. Chest.

[51] Chacko CJ, Gopal S (2015). Systematic review of use of β-blockers in sepsis. J. Anaesthesiol. Clin. Pharmacol..

[52] Liu H, Ding XF, Zhang SG, Wang HX, Luo YG, Duan XG (2019). Effect of esmolol in septic shock patients with tachycardia: A randomized clinical trial. Zhonghua Yi Xue Za Zhi.

[53] Wang Z, Wu Q, Nie X, Guo J, Yang C (2015). Combination therapy with milrinone and esmolol for heart protection in patients with severe sepsis: A prospective, randomized trial. Clin. Drug Investig..

[54] Wang S, Li M, Duan J, Yi L, Huang X, Chen D (2017). Effect of esmolol on hemodynamics and clinical outcomes in patients with septic shock. Zhonghua Wei Zhong Bing Ji Jiu Yi Xue..

[55] Xinqiang L, Weiping H, Miaoyun W, Wenxin Z, Wenqiang J, Shenglong C (2015). Esmolol improves clinical outcome and tissue oxygen metabolism in patients with septic shock through controlling heart rate. Zhonghua Wei Zhong Bing Ji Jiu Yi Xue..

[56] Kakihana Y, Nishida O, Taniguchi T, Okajima M, Morimatsu H, Ogura H (2020). Efficacy and safety of landiolol, an ultra-short-acting β1-selective antagonist, for treatment of sepsis-related tachyarrhythmia (J-Land 3S): A multicentre, open-label, randomised controlled trial. Lancet Respir. Med..

[57] Seymour CW, Liu VX, Iwashyna TJ, Brunkhorst FM, Rea TD, Scherag A (2016). Assessment of clinical criteria for sepsis: For the third international consensus definitions for sepsis and septic shock (sepsis-3). JAMA.

